# Investigations of airborne tire and brake wear particles using a novel vehicle design

**DOI:** 10.1007/s11356-024-34543-9

**Published:** 2024-08-28

**Authors:** Manuel Löber, Linda Bondorf, Tobias Grein, Sven Reiland, Steffen Wieser, Fabius Epple, Franz Philipps, Tobias Schripp

**Affiliations:** 1 German Aerospace Center (DLR), Institute of Combustion Technology, Pfaffenwaldring 38–40, 70569 Stuttgart, Germany; 2 German Aerospace Center (DLR), Institute of Vehicle Concepts, Pfaffenwaldring 38-40, 70569 Stuttgart, Germany

**Keywords:** Non-exhaust emissions, Ultrafine particles, Tire wear, Tire road wear particles, Scanning electron microscopy, Energy-dispersive X-ray spectroscopy, Microplastic, Urban air quality

## Abstract

Non-exhaust emissions have become an increasingly important issue as their levels continue to rise and the health effects of particulate matter (PM) are more widely discussed. To address this issue, a vehicle demonstrator with integrated emission reduction of tires and brakes was developed as part of the Zero Emission Drive Unit Generation-1 (ZEDU-1) project. This novel concept includes the removal of tire road wear particles (TRWP) with a strong ventilation/filtering system and an enclosed multi-disk brake, making it a suitable tool for the investigation of non-exhaust emissions. Particle number (PN) and particle size distribution (PSD) measurements down to 2.5 nm were performed on a chassis dynamometer and on a test track. Due to the low background concentrations on the chassis dynamometer, it is possible to distinguish between tire and brake wear and to characterize even a small number of particle emissions. It could be shown that about 30 % less particles are emitted by the vehicle, when using the novel multi-disk brake instead of the conventional brake. The highest TRWP emissions were collected during acceleration and harsh braking. Characterization of the collected particles using scanning electron microscopy (SEM) and energy-dispersive X-ray spectroscopy (EDS) revealed diverse particle shapes and differences between particles generated on the dynamometer and on a test track.

**Graphical Abstract:**

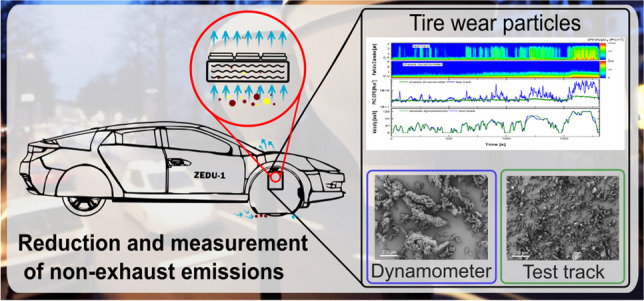

**Supplementary Information:**

The online version contains supplementary material on both test locations, various Fig. on size distribution, drum force, EDX spectra and CS results. Available at 10.1007/s11356-024-34543-9.

## Introduction

Particulate matter is considered a major global health risk, influencing the development of a variety of diseases, such as diabetes, hypertension, and cancer (Alemayehu et al. 2020). In the past, PM has been dominated by emissions from combustion processes. However, these emissions especially in road traffic are constantly decreasing, mainly due to the development of cleaner engines and the electrification of passenger cars (Harrison et al. 2021; Woo et al. 2022a). At the same time, particulate emissions from non-exhaust sources, such as road, brake, and tire wear, are steadily increasing due to the growing number and weight of vehicles, making them a major source of PM (Grigoratos and Martini 2015; Lin et al. 2022). Especially in larger cities, airborne particles originating from vehicular traffic are an important contributor to overall air pollution (Jandacka and Durcanska 2019).

In general, particles collected from the tire road interface consist not only of pure tire wear, but also of road wear (RW) and resuspended road dust, which in turn may consist of various types of suspended material such as tire, road and brake debris, exhaust emissions, and many more. To clarify that tire wear is always a mixture, the term tire road wear particles (TRWP) is recommended and used below, including those generated on the dynamometer (Zhang et al. 2023).

Previous studies indicated that the size distribution of brake wear emissions ranges from approximately 10–20 nm to 0.1–2 µm. For TRWP emissions, most particles are found to be in the 10–200 nm size range (Fussell et al. 2022). Especially, emissions from TRWP have diameters of less than 100 nm (Gustafsson et al. 2008), making them particularly dangerous due to their high reactivity and ability to easily penetrate the human body, where they can enter the bloodstream and thus spread throughout the body (Schraufnagel 2020).

Reducing brake and TRWP is therefore a key task in improving air quality and minimizing health risks. For this purpose, the ZEDU-1 project was launched, aiming for the construction of a vehicle demonstrator with strongly diminished non-exhaust emissions. In order to reduce brake wear, a fully encapsulated brake has been developed that does not release any wear particles. The optional switchable disk brake also made it possible to characterize brake wear emissions, taking advantage of the housing/ventilation and thus the corresponding sampling. Far more complex is the issue of reducing TRWP emissions. The scientific discussion of tire emissions is dominated by two terms: tire abrasion (Sommer et al. 2018), and airborne TRWP (Baensch-Baltruschat et al. 2020; Kim and Lee 2018).

Tire abrasion is considered the total amount of tire wear released into the environment and is a major source of microplastics, causing various environmental issues (Baensch-Baltruschat et al. 2020). It is estimated that more than 1.3 million tons of tire wear are released into the environment in the EU every year (Wagner et al. 2018). Most of the mass of the tire abrasion is concentrated in larger particles or chunks. A comprehensive study by the German Automobile Club (ADAC) found a wide range of tire wear over a mileage of 15,000 km. For a given tire size (e.g., 205/55 R16), the study found a wear rate between 82 and 151 g per 1000 km, demonstrating the variety of tire emissions (ADAC 2022).

Microplastic is considered to consist of polymer particles smaller than 5 mm. While the lower size limit varies in the literature, particles smaller than 0.1 µm are often referred to as nanoplastic (Hartmann et al. 2019). Although rubber, the main material of tires, is excluded from the ISO definition of plastic, some broader definitions that include rubber are also common (Verschoor 2015). Within this broader definition, tire microplastics (TMPs) play an important role in overall microplastic pollution and are considered the second largest primary source of microplastics in the oceans (Boucher and Friot 2017). TMPs are released not only from TRWP that are washed off roads or transported by wind, but also from recycled tire crumb (RTC) and tire repair-polished debris (TRD) (Luo et al. 2021). Airborne particles do not contribute significantly to the total tire abrasion, but are more abundant in PN and can cause the aforementioned health issues. Hence, a reduction of those airborne PM is crucial especially in urban areas. Unlike brakes, tires cannot be completely enclosed and therefore always are in an open system.

It is well known that experimental data on TRWP emissions is limited, as many studies are based on modeling or processing of existing older data. Therefore, the study of TRWP generation and characterization is crucial (Mennekes and Nowack 2022). However, appropriate sampling techniques are not well established. Sampling of tire particles consists often of filter sampling followed by pyrolysis gas chromatography/mass spectrometry (GC/MS) analysis (Panko et al. 2013; Youn et al. 2021). Different methods of generation of TRWP, such as formation by volatilization of the tire tread in a heated reaction chamber, are also investigated (Park et al. 2017). In addition, the generation of TRWP using a tire simulator (Dalmau et al. 2020; Kim and Lee 2018), a homemade test rig (Chang et al. 2020), or a specialized test vehicle (Tonegawa and Sasaki 2021), is also a common approach. On-line sampling during driving is mostly performed by attaching a tube close to the tire and testing the vehicle on a chassis dynamometer (Li et al. 2023), or on a real road (Lee et al. 2013). More sophisticated setups with disk brake encapsulation allow separate investigation of tire and brake wear (Feißel et al. 2024). Since many different parameters affect the formation of TRWP, such as temperature, load, and tire type, a detailed understanding of the formation processes is essential (Schläfle et al. 2023). TRWP are not easy to distinguish from urban background and resuspended road debris using on-line particle number concentration (PNC) and PSD measurements. SEM–EDS analysis is therefore a suitable tool to identify the elemental fingerprint of each particle as well as to characterize the particle morphology, since available particle sizers assume a specific shape (optical, aerodynamic, electromobility diameter) (Rausch et al. 2022).

Inspired by the many studies describing potential formation mechanisms and ways to investigate the TRWP generation, the goal of ZEDU-1 was to capture the full range of airborne TRWP, as well as reduce microplastic pollution. This was done by enclosing the tires as much as possible and removing the generated emissions by means of strong ventilation through a two-stage filter system. This tire housing and ventilation system can not only reduce emissions, but can also be used as a sampling system to study brake and tire emissions on the chassis dynamometer and on the road, as demonstrated in this study. The technical details of the demonstrator are covered in another publication (Wieser et al. 2022).

## Materials and methods

### Vehicle demonstrator and sampling positions

**Fig. 1:**
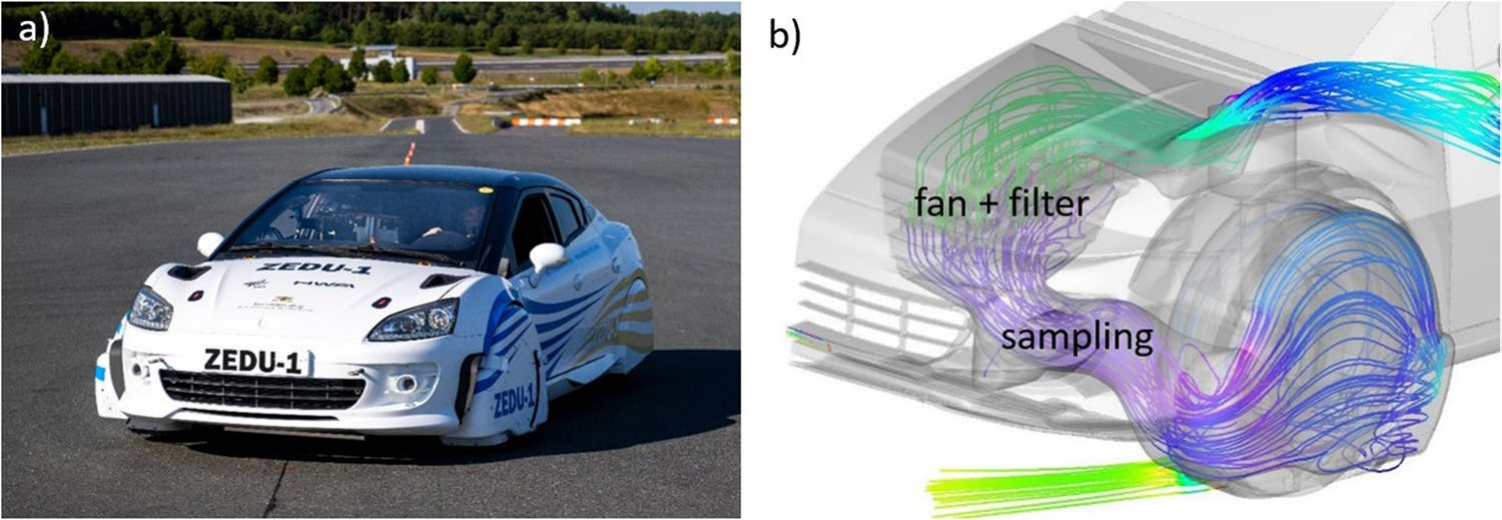
**a** ZEDU-1 demonstrator on the test track in Boxberg. **b** Sampling position for emission measurements. The simulated airflow path is displayed with colored lines indicating different pressure zones. **b** Reprinted (adapted), with permission, from (Wieser et al. 2022) © [2023] IEEE

The ZEDU-1 vehicle demonstrator, as shown in Fig. 1a, was developed within the ZEDU-1 project at DLR and constructed by HWA AG as a subcontractor with the goal of reducing non-exhaust emissions. The chassis of the demonstrator is based on a vehicle with a combustion engine from the used car market, which has been converted into a battery-electric vehicle. A specifically designed drive unit, consisting of an electric motor, planetary gear, and a multi-disk brake, has been integrated and patented (Kühner 2022). The oil-cooled multi-disk brake system captures all wear debris in the operating fluid, thus no brake emissions are emitted into the environment. Figure 1b shows the tire enclosure, as well as the suction and filtration system. The TRWP are drawn in at the tire contact surface below the housing. From there, they are passed through a pre-filter, the fan matrix, and the main filter. Finally, the cleaned air is released through openings in the engine hood. The fan matrix consists of twelve individual fans generating an air flow dependent on the vehicle speed up to a maximum of 250 l/s. The disk brake is normally not in use, but is still mounted and can be activated by a manual switch (otherwise, the piston is pushed back to prevent any friction). Since this disk brake is located inside the tire housing and is not additionally enclosed, all brake wear (if the disk brake is activated) are drawn into the fan/filter sampling system, similar to the TRWP emissions.

Particle sampling is located in front of the fan/filter system. The air particulate sampling was equipped with a gooseneck and a sampling nozzle, the diameter of which was adapted to the selected air flow. A carbonized tube was attached to each gooseneck and led into the interior of the vehicle. The sampled air was divided by flow splitters and connected to the inlets of the measuring devices by carbonized tubes.

The tests were conducted on the DLR chassis dynamometer and on the Bosch test facility in Boxberg, Germany. A detailed description of both test systems is provided in the Supplementary Information (SI).

### Aerosol measurement equipment

For measuring the PNC from 2.5 nm to 10 µm as well as the PSD between 5.6 nm and 10 µm, different particle measurement techniques were used. The measuring equipment was placed inside the vehicle, with the co-driver seat removed for this purpose. A 12.8 V, 400 Ah lithium-ion battery pack (Victron Energy), and a 230 V inverter were also installed in the vehicle for the test facility measurements. All measuring instruments operate at 1 Hz.

A TSI 3776 Condensation Particle Counter (CPC) was used to quantify the total PN for diameters between 2.5 nm (D50) and 3 µm on the chassis dynamometer. For test track measurements, two Brechtel Mixed Condensation Particle Counter (MCPC) were included in the setup, since they are less sensitive to tilt and rapid motion. The MCPC counts airborne particles from 7 nm (D50) to 2 µm. A TSI 3090 Engine Exhaust Particle Sizer (EEPS) measures the size distribution of aerosol particles between 5.6 and 560 nm via 32 channels. Larger particles from 300 nm to 10 µm are detected with a TSI 3330 Optical Particle Sizer (OPS) in 16 size-selective channels. The ratio of volatile and solid particles was determined using a combination of a CPC and a catalytic stripper (Catalytic Instruments, CS015). A correction has been applied to the data provided by the CS to consider the thermophoretic and diffusion losses according to the Application Note CI-0009 of Catalytic Instruments.

The instrumentation is calibrated frequently by the manufacturer. The particle losses within the sampling system were determined by sodium chloride (NaCl) salt particles generated by a portable test aerosol generator (TSI, Model 3073). An overview of the performed tests is given in Table 1.

**Table 1:** Overview of test cycle, test location, measurement instrumentation, and the covered particle size range for each figure. displayed in this study

Test cycle	Test location	Instrumentation	Size range	Fig. no
ZEDU Brake^1^	Chassis dynamometer	CPC 3776, EEPS	2.5 nm–3 µm	Fig. 2
WLTC Class 3b	Chassis dynamometer	CPC 3776, EEPS, ELPI, OPS	2.5 nm–10 µm	Fig. 3, Fig. 4
WLTC Class 3b	Test track	MCPC, EEPS, OPS	5.6 nm–10 µm	Fig. 3, Fig. 4

^1^DLR intern cycle, specifically designed for investigation of brake and tire wear

TRWP are collected using an Electrical Low-Pressure Impactor (ELPI +, Dekati) on 13 size-separated stages from 16 nm to 10 µm for testing on the chassis dynamometer. Polycarbonate foil is used as the substrate for particle deposition, and the ELPI + charger is turned off during collection in order to reduce particle losses in the corona charger. The foils were analyzed via scanning electron microscopy using an Ultra Plus from Carl Zeiss AG.

Furthermore, particles captured by the pre-filter on the chassis dynamometer and on the test track are analyzed by SEM–EDS. For this purpose, a piece of the filter was cut out and placed in an ultrasonic bath with isopropanol for 10 min. Afterwards, the samples were transferred to a conductive carbon pad and coated with a 20 nm layer of platinum using a Quorum 150 V plus sputter coater. The elemental composition of the particles was determined using an EDS detector (Ultim Max, Oxford Instruments). Platinum was excluded from the quantification by the manufacturer’s software. Since quantification of light elements is difficult with EDS (especially for particles), no numbers were added to the spectra. Instead, the ratios of the identified elements were plotted as bars based on their weight %.

## Results and discussion

### Brake and TRWP emissions

In standard operation, the ZEDU-1 demonstrator uses the aforementioned multi-disk brake, which is fully encapsulated and therefore emission-free. However, the original disk brake is still mounted on the front axle of the demonstrator and can be switched on or off as required for emission tests. This enables the sampling of combined tire and brake wear emissions, as well as the exclusive measurement of tire emissions. A cycle consisting of five braking events with increasing brake deceleration, called ZEDU brake (Bondorf et al. 2023), was used to determine whether tire and brake wear can be distinguished when a mixture of the two types of particles is present in on-line measurements.

**Fig. 2:**
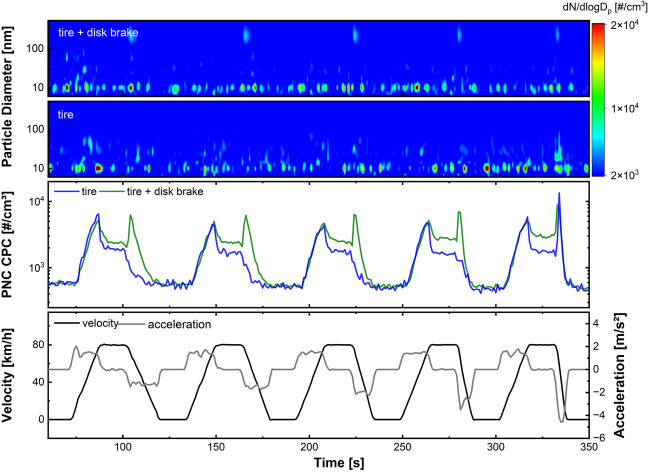
Comparison of a cycle driven with disk brake and without on the chassis dynamometer. The green curve refers to the CPC data using the disk brake; the blue curve corresponds to the CPC data using the multi-disk brake, showing only the tire emissions. The upper EEPS contour plot corresponds to the green curve, the lower one to the blue curve. In addition to the driven velocity (black), the acceleration is displayed on the right y-axis (gray), to emphasize the increase in deceleration

This brake test cycle on the chassis dynamometer and the corresponding emission curves are depicted in Fig. 2. The vehicle velocity is shown in black and the corresponding acceleration is plotted on the right y-axis in gray to emphasize the increasing deceleration. Both emission curves were recorded on the dynamometer during two comparable driving cycles. The blue graph shows the emissions of TRWP only, while the green curve represents the combined emissions from tire and brake wear. Therefore, any additional emissions visible in the green curve can be attributed to brake wear. In the tire-only tests (blue curve), there is a strong emission peak upon acceleration of all five segments, followed by a period of constant emissions. During deceleration, the concentration drops rapidly. Only during the last, strongest braking, a peak of higher TRWP emissions is visible, which is triggered by the strong braking and presumably leads to the tire slipping. The acceleration peak of the green curve fits well with those recorded with the tire only. At a constant speed, the concentration is higher and increases with each segment. The reason for this is the slippage of the brake pads, which causes emissions even when the brake pedal is not depressed. This is a common phenomenon, which is why the brake cylinders were pushed back for the TRWP tests. During deceleration, a peak is recorded for all five segments, which becomes narrower as the deceleration becomes stronger and thus shorter. The additional emissions, when comparing the blue and the green curve, can be assigned to brake wear and the PNC (tire + brake) are roughly 30 % higher compared to tire emissions.

The PSDs are depicted in the lower (tire) and upper (tire + disk brake) contour plots of Fig. 2. In both plots, a mode at around 10 nm is visible during the whole cycle. Additionally, the lower plot shows a second mode during the last braking event (330–340 s) between 10 and 100 nm. The upper plot shows, in addition to the less pronounced second mode around 50 nm, a third mode with a slightly larger magnitude at about 200 nm during braking (105 s, 166 s, 225 s, 280 s, 334 s). For a better analysis, both PSDs of the last breaking are shown in Fig. S1. The higher tire emissions during the last deceleration (334 s) could be explained by a higher negative force recorded on the dynamometer drum (− 4600 N), reached in a much shorter time (steeper slope) than the corresponding force recorded for the disk brake test (− 3700 N). The higher friction applied to the tire therefore results in higher emissions. The force and corresponding velocity recorded for the last braking event on the chassis dynamometer are depicted in Fig. S2.

It was shown that brake wear emissions generated on the chassis dynamometer can be clearly identified and distinguished from TRWP emissions due to their different size distribution and the low background concentration on the chassis dynamometer. Five different braking events, ranging from light to hard braking, showed an average increase in PNC of about 30 % compared to only TRWP emissions when using the multi-disk brake. Comparative braking tests conducted on the test track were overlapped by the higher background concentration and higher TRWP emissions.

### Tire road wear particle emissions

TRWP emissions were investigated on the chassis dynamometer and on the test track using the Worldwide harmonized Light Duty Test Cycle Class 3b (WLTC). To protect the battery from damages, the top speed was limited to about 120 km/h, slightly slower than the WLTC requirement (131 km/h). To investigate tire and no brake wear emissions, all tests were performed with the built-in multi-disk brake.

**Fig. 3:**
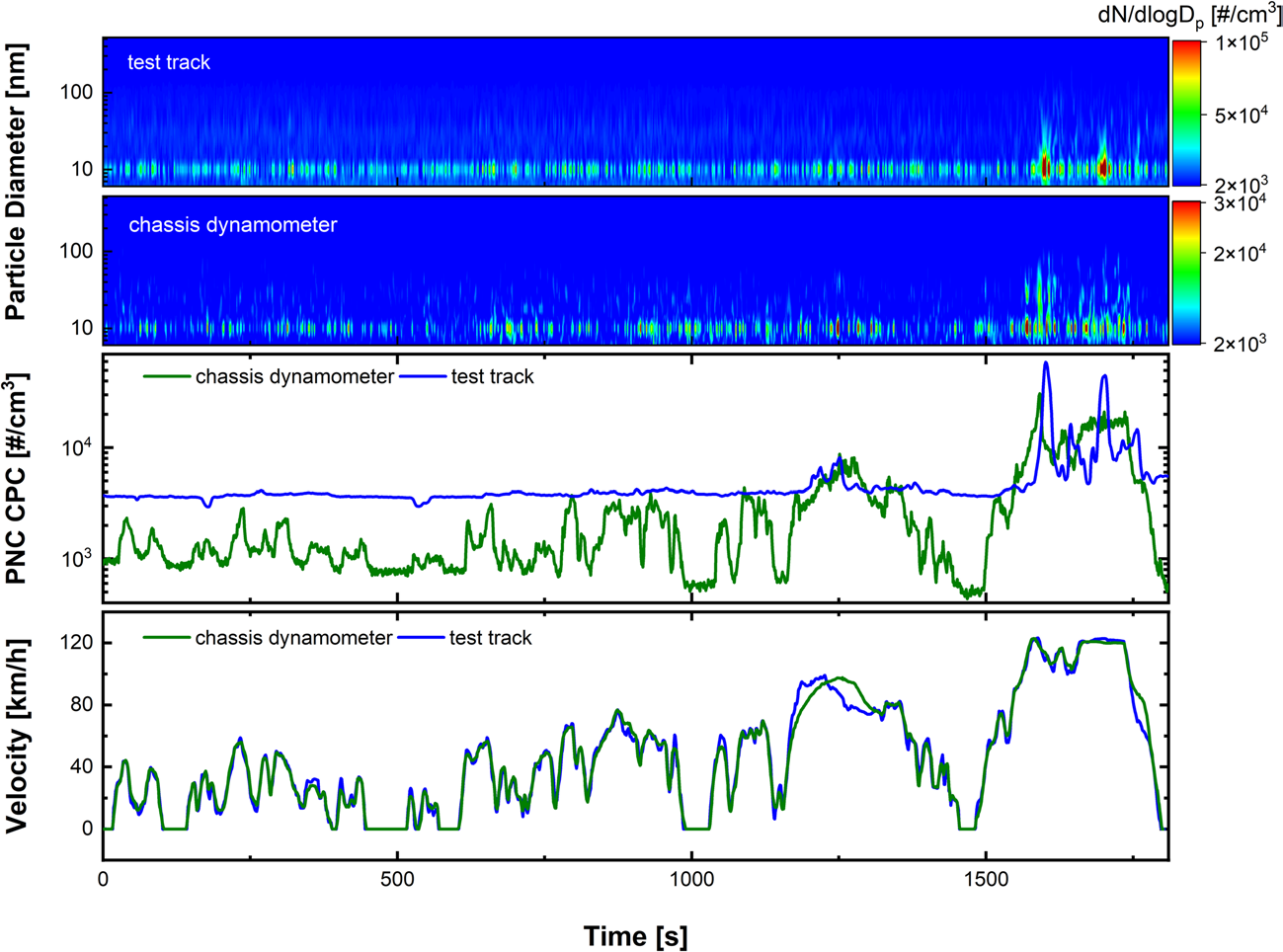
WLTC recorded on the chassis dynamometer and on the test track in Boxberg, Germany. The green curves refer to the data from the chassis dynamometer; the blue curves correspond to the data from the test track. The lower EEPS contour plot corresponds to the green curve, the upper to the blue curve

The PNC and the corresponding PSD are depicted in Fig. 3. At both test sites, the emissions were measured at the sampling locations of the pre-filter. The green curve (measured on the chassis dynamometer) follows the shape of the vehicle velocity, with PNCs being below 4 × 10^3^ #/cm^3^ in the first two parts of the cycle (low and medium velocity, 0–1030 s) and higher concentrations (8 × 10^3^ and 3 × 10^4^ #/cm^3^) in the last two segments (high and extra high velocity, 1030–1800 s). In particular, a strong peak appears at the end of the cycle (at 1600 s), which can be assigned to the acceleration of the vehicle. The background concentration (sections where the vehicle stops) varies between 5 × 10^2^ and 1 × 10^3^ #/cm^3^, which is much lower than in traffic-rich or even rural regions (von Schneidemesser et al. 2019).

The corresponding size distribution is depicted in the lower contour plot in Fig. 3 and shows that particles with diameters around 10 nm are present throughout the cycle. During the last segment (extra high velocity, 1480–1800 s), a second mode of particles with diameters between 10 and 50 nm is visible, which is strongest at the acceleration peak at about 1600 s. The PNC recorded on the test track (blue curve) is generally higher and the background concentration is about 4 × 10^3^ #/cm^3^. Therefore, at the two lower speeds (low and medium velocity, 0–1030 s), no specific emission events are visible since they are overlapped by the high background. However, the last two sections (high and extra high velocity, 1030–1800 s) provide particle emission that clearly stands out from the background (about 9 × 10^3^ #/cm^3^). In the last segment (extra high velocity, 1480–1800 s), two strong peaks are visible; the first one occurs during acceleration (at 1600 s) with a PNC of about 6 × 10^4^ #/cm^3^; the second one appears during constant velocity (at 1700 s) with a PNC of roughly 4.5 × 10^4^ #/cm^3^. The first peak (at 1660 s) is similar to the one that occurs on the chassis dynamometer caused by the acceleration to the high velocity (approximately at 120 km/h). The second peak (at 1700 s) is not observed on the chassis dynamometer tests (green curve). A possible explanation for the second peak is that a local temperature increase triggers the generation of small particles, since the OPS data, as discussed later, do not show an increased PNC around 1700 s. The corresponding PSD is displayed in the upper contour plot of Fig. 3 and shows a similar yet more constant mode around 10 nm. However, when the two peaks appear (1600 s and 1700 s, respectively), a much higher concentration of 10 nm particles is visible. There is also a broader size distribution of 6 to 50 nm around these peaks and a second larger mode between 60 and 90 nm. For a closer examination of the acceleration peaks, a mean size distribution during the acceleration phase is plotted in Fig. S3. The particles collected on the test track are slightly larger than those on the chassis dynamometer. This size difference could be explained due to cornering on the test track, since the tests were conducted on an oval-shaped track. This is in line with previous studies that indicated differences in size distribution on a proving ground (60–70 nm) and a road simulator (30–40 nm) determined at different constant speeds (Kwak et al. 2014). In general, emissions increase with higher speeds, as reported in the literature (Kim and Lee 2018). Higher TRWP emissions during acceleration are reported to occur only under race start conditions and are thought to be due to tire slip (Mathissen et al. 2011).

**Fig. 4:**
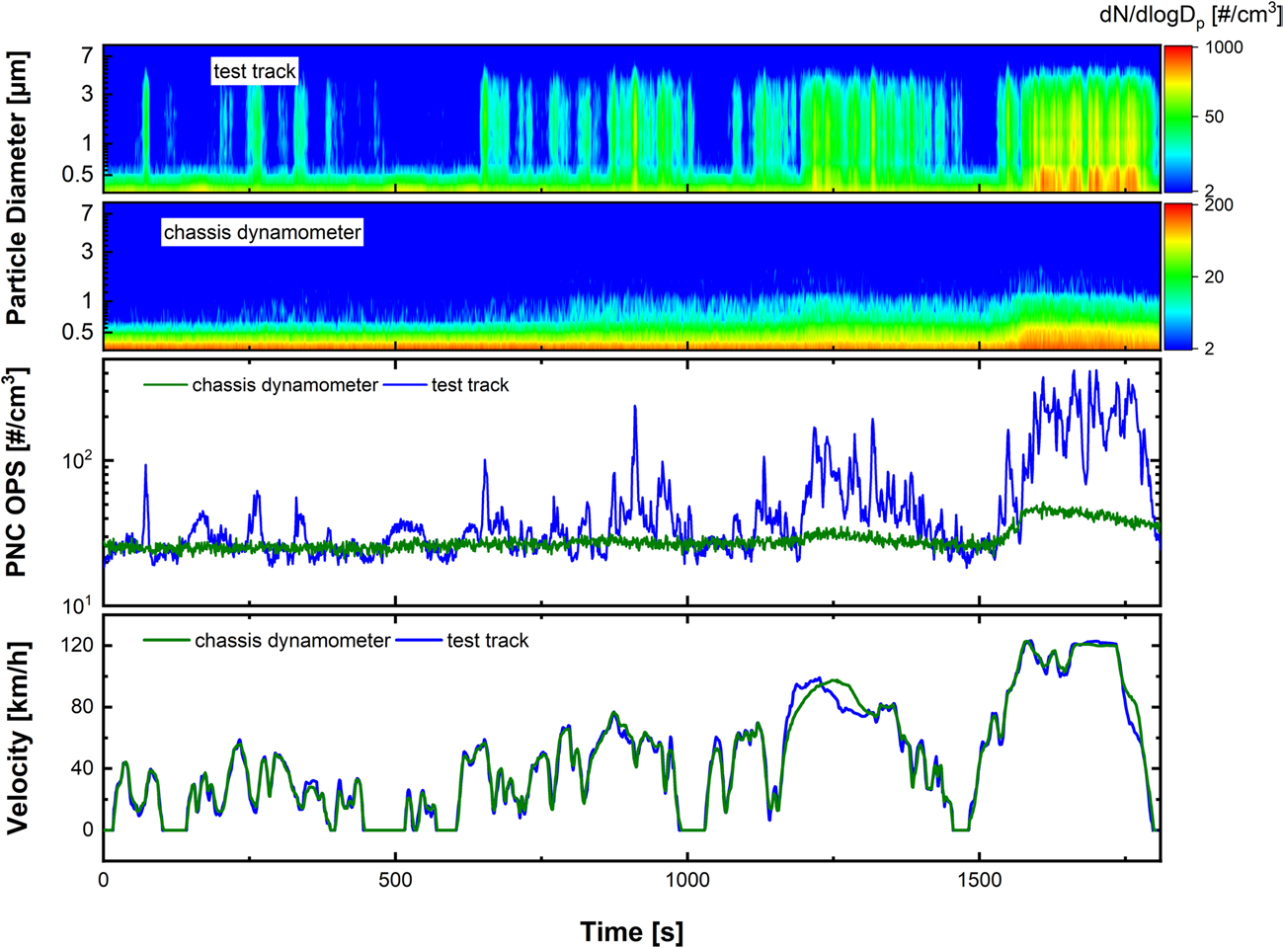
Comparison of PNC and PSD recorded with an OPS on the test track and on the chassis dynamometer

Figure 4 shows the OPS data for particles with a diameter between 300 nm and 10 μm, which was also recorded on the pre-filter sampling position on the chassis dynamometer (green) and on the test track (blue) using the WLTC. On the chassis dynamometer, the PNC is at a constant concentration of roughly 20 #/cm^3^. An increase in the total number of particles with increasing velocity is visible, similar to the CPC data in Fig. 3. However, this is only the case for the two high-velocity segments (high and extra high velocity, 1030–1800 s), where the concentrations are around 30 #/cm^3^ (high velocity, 1030–1480 s) and 45 #/cm^3^ (extra high velocity, 1480–1800 s) respectively. In the lower contour plot, it becomes visible that these are mainly particles with a mode around the lower detection limit of 300 nm, since the number of particles decreases successively in the larger channels. On the test track, the total number of particles is more variable. However, a tendency towards increasing numbers at higher velocities can be seen. At the first and lowest velocity (0–500 s), only a few weak peaks appear with concentrations between 50 and 100 #/cm^3^. At the second and third segments (medium and high velocity 500–1480 s), more peaks, some above 100 #/cm^3^, arise. At the last extra high velocity (1480–1800 s), concentrations above 400 #/cm^3^ are visible. In the contour plot, the broader size range of the particles becomes visible down to the micrometer range. Significantly larger particles are sucked in than during the chassis dynamometer tests. The averaged PSD of the last segment is shown in the SI (Fig. S4). The constant PNC recorded on the dynamometer seems to be within the background noise, since the same concentration was recorded before the start of the test. However, the increase in PNC at higher speeds (at about 1200 s and 1600 s) is thought to be accounted for by real TRWP emission, since the PNC also increases on the test track during the same high velocity segments.

The particles drawn into the ZEDU-1 demonstrator differ depending on the test system. Ultrafine particles, especially in the 10 nm range, occur for both test systems and show a very similar emission curve in the driving cycle. This indicates that the emissions are caused by the tire, e.g., by thermal processes. With regard to larger particles between 300 nm and 10 µm, there are stronger differences in the test systems. This could be caused by the road surface, temperature differences, or by resuspension of road dust.

### TRWP emission factors

Emission factors (EF) for the PNC (CPC) of the TRWP were determined from the WLTC performed on the dynamometer and test facility, respectively. For this purpose, the background concentration was subtracted from the total PNC and then multiplied by the recorded air flow volume. At the chassis dynamometer, an EF of 2.1 × 10^10^ #/tire km was found for the right front tire. This value can be multiplied by a factor of 2 for the front axle. The forces recorded at the rear axle are much lower than such recorded at the front axle. Hence, much lower emissions are expected that do not have a major effect on the overall emission factor which might account for 20 % of the vehicles’ whole TRWP emissions.

The values determined on the test track are around 1.0 × 10^10^ #/tire km. Although single peak events at the test facility produce much larger emissions than at the chassis dynamometer, the overall emissions during the WLTC cycle seem comparable, at least for particles in the range from 7 nm to 2 µm. However, most of the emissions recorded at the test facility are overlapped by the large background, and thus not considered for the EF. The OPS emissions (0.3–10 µm) recorded at the test facility are higher than those recorded at the chassis dynamometer, which are 4.7 × 10^7^ #/tire km determined at the dynamometer and 3.0 × 10^8^ #/veh km determined at the test facility for the front axle respectively.

These values are lower than some of those reported in the literature. Dahl et al. reported 3.8 × 10^11^ #/veh km on a quartzite surface at a constant speed of 50 km/h at a particle size range of 15–700 nm (Dahl et al. 2006). A reason for the higher value reported in literature could be that cornering was not considered at the chassis dynamometer, which is known to have a large impact on the overall TRWP emission. The WLTC was performed at an oval high-speed track with also little cornering.

### REM-EDS analyses

**Fig. 5:**
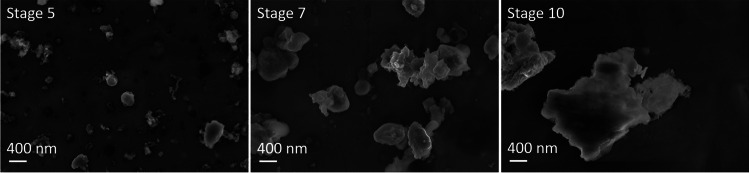
SEM micrograph with Inlense detector at an EHT of 5 kV of particles collected by the ELPI + on the chassis dynamometer

Figure 5 shows particles collected in front of the filter with the ELPI + during a WLTC cycle on the chassis dynamometer. According to the calibration, particles with a mean aerodynamic diameter of approximately 96 nm, 257 nm, and 950 nm are collected at stages 5, 7, and 10. Stage 5 shows nearly spherical particles of 100–200 nm in size. These rounded particle shapes are most likely formed from several gaseous molecules that condensed rapidly after their formation. By stage 7, they have clumped together to form larger structures, up to several microns in diameter. At stage 10, larger particles show a different morphology with relatively sharp edges instead of rounded shapes, suggesting an abrasive and mechanical formation process. The gas-phase origin of the smaller particles is consistent with experiments using a catalytic stripper, where a WLTC at 30 °C (no catalytic activity) and 350 °C (highest catalytic activity) was performed that showed a significant decrease in PNC at the higher temperature. The plot is shown in Fig. S5. Moreover, the literature describes the appearance of local hot spots, where the evaporation of volatile organic compounds to form nanoparticles takes place (Woo et al. 2022b). Furthermore, lab studies found an increase in volatile particles formed at temperatures above 160 °C (Park et al. 2017).

To study TRWP captured in the filter on both the chassis dynamometer and the test track in Boxberg, samples were investigated using SEM and EDS. A comparison of an unused and a used filter is shown in the SI (Fig. S6), emphasizing the amount of material captured. An EDS spectrum of an unused tire tread sample was analyzed and found to contain C, O, Si, S, and Zn. Rubber particles in the mm range were collected in front of the filter and are shown in Fig. S7, demonstrating the potential to remove also larger microplastic particles.

**Fig. 6:**
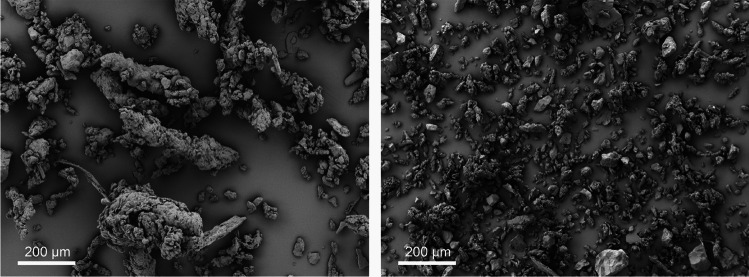
SEM micrograph of particles collected on the chassis dynamometer (left) and on the road (right). The figures were taken at an EHT of 5 kV (left) and 3 kV (right) with the SE2 detector

**Fig. 7:**
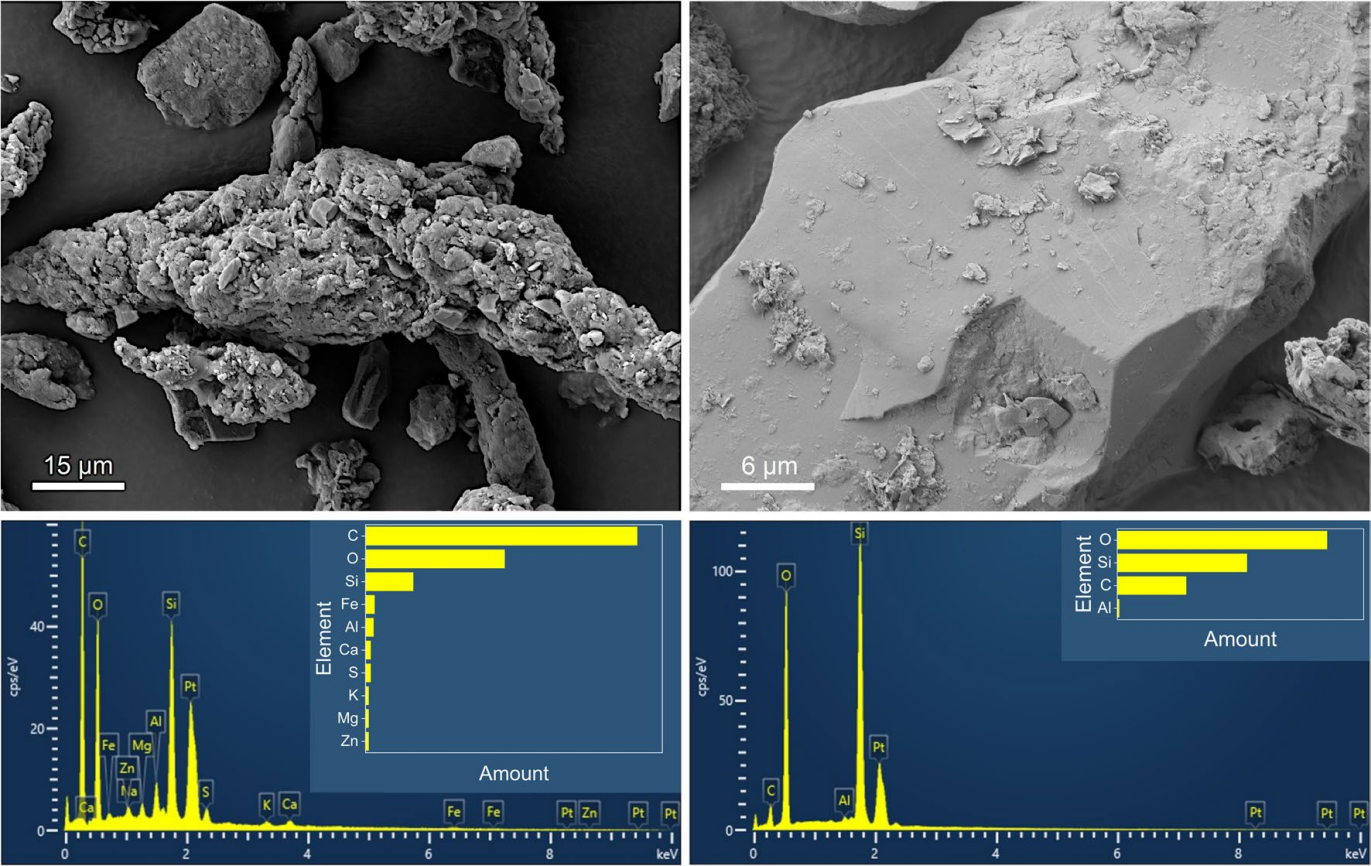
Higher magnification of a TRWP particle (left) and a road wear particle (right) both with their corresponding EDS spectra. The images were taken at an EHT of 10 kV (left) and 3 kV (right) with the SE2 detector. The EDS spectra were recorded at 10 kV respectively

**Fig. 8:**
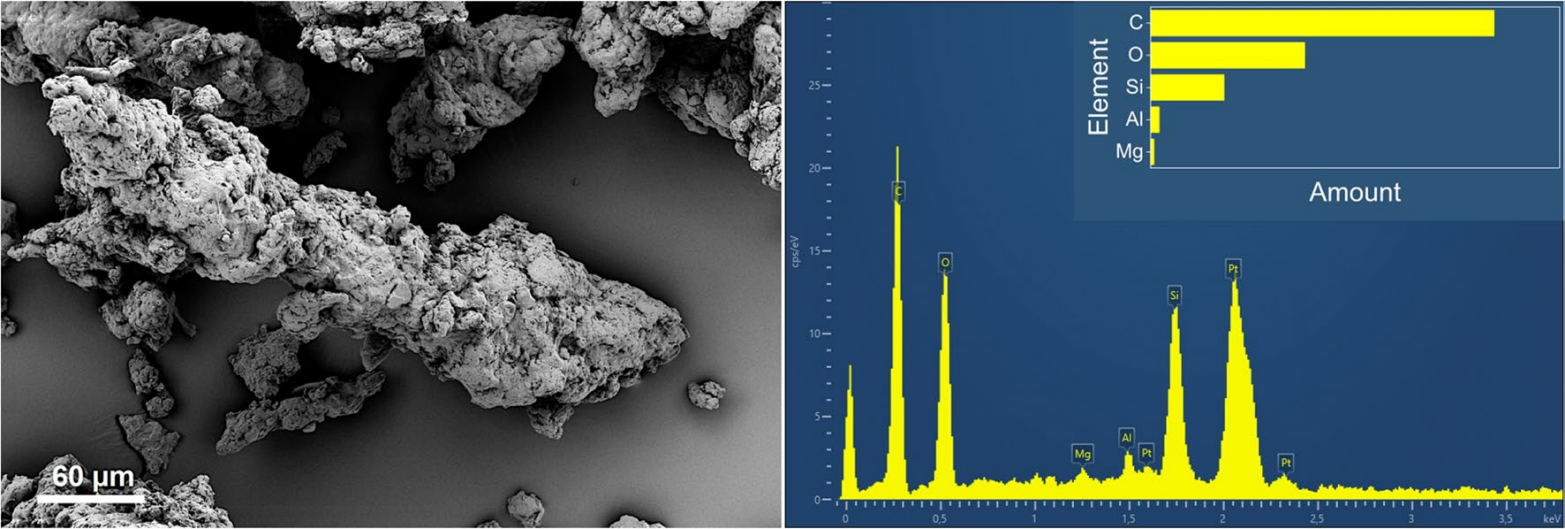
SEM micrograph of particles collected on the chassis dynamometer (left), taken at 5 kV with the SE2 detector, and EDS spectrum of the largest particle, visible in the center of the left image (right), recorded at an EHT of 7.5 kV

Figure 6 shows an overview magnification of particles collected on the chassis dynamometer (left) and on the test track (right). Many particles with a coiled elongated shape can be seen. However, the particles generated on the chassis dynamometer are significantly larger than those generated on the road surface (test track). Beside those coiled particles, more compact grains with a smooth surface can be spotted on the right part of Fig. 6. Information about the origin of these particles is provided by an analysis with EDS. A spectrum of a coiled TRWP collected on the test track is depicted in Fig. 7 (left). All five elements (C, O, Si, S, and Zn) of the tire tread can be detected. Apart from carbon, oxygen, and silicon, the elemental distribution is quite heterogeneous. The detection of the important tire elements sulfur and zinc is not always possible, since the fraction in the tire tread is comparatively low, so that a small depletion (e.g., by thermal alteration) is sufficient to be below the detection limit. In addition, most of the particles are covered by a layer of road dust, which makes detection even more difficult. As also shown in Fig. 7, some of the metals Fe, Al, Ca, Mg, and K are also present in smaller quantities. These elements are described in the literature to occur in the pavement (Alves et al. 2020). A different type of particle is visible on the right part of Fig. 7. It has a smoother texture and a more compact appearance than the particle shown on the left. The corresponding EDS spectrum shows high amounts of Si and O, and thus presumably SiO_2_, which as quartz grains is a common component of asphalt. This type of particle is therefore RW that has been mechanically abraded from the road surface. The appearance of the elongated TRWP with smaller grains attached to their surfaces that originate from the road pavement is supported by the literature (Gehrke et al. 2023). Particles collected on the chassis dynamometer have much fewer grains on their surface as visualized in Fig. 8 (left) and contain predominantly oxygen, carbon, and silicon (see Fig. 8 right). In contrast to the particles collected at the test facility, the cleaner surface of the dynamometer drum resulted in much fewer elements being identified. Therefore, the main tire constituents C, O, and Si are mainly present. Other elements, such as Al or Mg, are found only in trace amounts and could be due to some dust deposited on the drum. Chemical analysis is therefore difficult because the road and TRWP are mixed during the wear process. In particular, the samples from the test facility showed a large amount of road debris that was deposited with the TRWP. The proportion of RW in TRWP is reported to be approximately 60 %, derived from tunnel samples (Wagner et al. 2024). From focused ion beam (FIB) SEM studies, it is known that the smaller RW particles are also present inside the larger TRWP, supporting the formation by coiling (Rausch et al. 2022). The finding of larger wear particles generated on the chassis dynamometer can be explained by either the difference in pavement or the temperature of the hot air and road surface during the measurements on the test track.

## Conclusion

This study describes a novel vehicle concept to investigate and characterize brake wear and TRWP emissions. The conventional disk brake is replaced by an emission-free multi-disk brake. However, the difference in emissions could be investigated by switching between the multi-disk brake and the conventional brake. It was found that at least 30 % of total non-exhaust emissions can be saved with the new brake concept. To reduce TRWP emissions, and also to study both tire and brake wear, a matrix of fans generating up to 250 l/s of air flow sucks particles out of a tire housing through a two-stage filter system, removing most of the particles and releasing the filtered air into the environment. This not only removes the airborne fraction (mainly PM10), but also eliminates the larger chunks that are a few millimeters in size, thus reducing the environmental impact of the tire microplastics. Comparative driving tests on a chassis dynamometer and on a test track showed similar PNC. While the PSD on the test bench is between 6 and 50 nm, the data from the test track showed an additional larger mode between 60 and 90 nm, which is presumably due to lateral acceleration. In the coarse size range up to 10 µm, only particles less than 1 µm in diameter were observed on the dynamometer, whereas two larger modes around 1 and 1.5 µm were detected on the test track. This could be additional resuspended road dust that is not (or less) present on the chassis dynamometer. Moreover, the higher temperature during the test track tests, with a surface temperature of more than 60 °C, could affect the mechanical formation process of wear towards larger particles. Material collected on the pre-filter during the dynamometer and test track tests was analyzed by SEM/EDS. Particles collected on the dynamometer were elongated and coiled, and most were several 100 µm in length. Particles gathered on the test track had a similar appearance, but were much smaller (less than 100 µm). In addition, many smaller grains are attached to the surface of the coiled TRWP, showing a greater amount of deposited material in contrast to the clean dynamometer surface. Furthermore, plenty of compact particles with a fine surface texture are observed on the test track sample. EDS analysis showed that these fine textured grains consist of mostly silica, and can therefore be attributed to RW. However, a detailed chemical assignment to a specific particle type seems to be difficult with EDS, since the particles are covered by a layer of road/surface dust, resulting in a general distribution of most elements.

Experiments performed on a chassis dynamometer and on a test track showed that a combination of both test scenarios is the best choice for a comprehensive investigation of non-exhaust emissions. When investigating emissions, but also when developing new methods for reducing emissions, it is important to consider the entire size spectrum of airborne particles, as there are considerable size-dependent differences. Particle number concentration and size distribution provide important information on particle formation. It also became apparent that different effects on particle formation, such as surface textures, surface dust, cornering, and temperature, need to be investigated in more detail, which will be subject of a future study.

## Supplementary Information

Below is the link to the electronic supplementary material.

Supplementary file1 (DOCX 942 KB)

## Data Availability

The relevant data from this research are available in the authors’ repositories.
