# Supplementary material for: Investigations of airborne tire and brake wear particles using a novel vehicle design
Source: Environ Sci Pollut Res Int. 2024 Aug 28;31(40):53521–31. doi: 10.1007/s11356-024-34543-9 (PMC11379764; doi:10.1007/s11356-024-34543-9)
Supplement: Supplementary file 1 — Supplementary file1 (DOCX 942 KB) [file 11356_2024_34543_MOESM1_ESM.docx]

**SUPPLEMENTARY INFORMATION**

Investigations of Airborne Tire and Brake Wear Particles Using a Novel Vehicle Design

Manuel Löber^a^,^*^,^#^, Linda Bondorf^a^,^#^,Tobias Grein^a^, Sven Reiland^b^, Steffen Wieser^b^, Fabius Epple^b^, Franz Philipps^b^, Tobias Schripp^a^

^a^German Aerospace Center (DLR), Institute of Combustion Technology, Stuttgart 70569, Germany

^b^German Aerospace Center (DLR), Institute of Vehicle Concepts, Stuttgart 70569, Germany

MATERIALS AND METHODS

**Chassis dynamometer**

The chassis dynamometer has four independently driven 48" rollers with 100 kW continuous power each, which allows to simulate driving conditions for front-, rear- or all-wheel drive concepts as well as for hybrid vehicles. The coating of the rollers is a rough material optimized for reduced slippage. The vehicle drives can be used for all common and specially defined cycles, including gradients. The chassis dynamometer is integrated into a climatic chamber that can be operated at various temperatures from - 40 °C to + 60 °C, humidities from 0 % to 80 % at the positive temperature range and circulating air volumes as well as a variable wind simulation up to 120 km/h. For this study a constant temperature of 23 °C and relative humidity of 20 % was applied. While driving the airstream was proportional to the driving velocity. The drums adherent layer consists of a CrNiMo alloy. The top layer is composed of a FeCrB alloy, with a surface roughness of 114 µm.

**Test facility**

The Bosch test facility in Boxberg, Germany, was used to perform realistic driving scenarios. The test facility consists of real road surfaces that are cleaned daily. Many different tracks are available, allowing driving scenarios such as high speeds, cornering and uphill driving. The tests were carried out in summer during a heat wave in which air temperatures of between 35 °C to 38 °C and roadway temperatures of more than 60 °C were measured.

RESULTS AND DISCUSSION

**Brake and tire wear emissions**


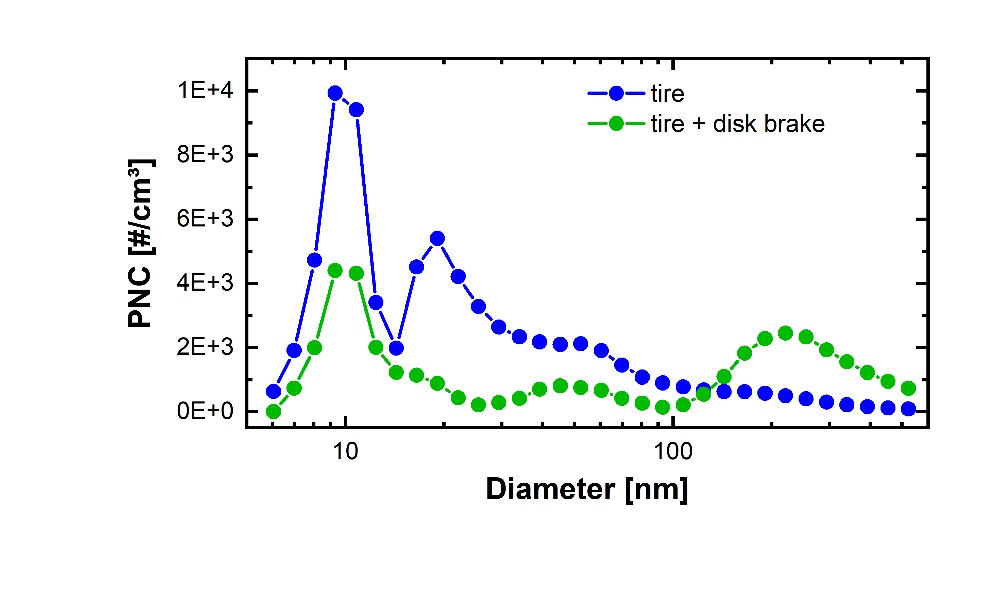


**Fig. S1** PSD derived from EEPS data of the emissions recorded with disk brake and with multi-disk brake on the test track. The values are means of PNCs recorded between 330 s and 340 s (last brake event in Fig. 2)

The values of Fig. S1 are derived from the last braking event in Fig. 2 (330 s – 340 s). Here the two modes (10 nm and 20 nm) recorded during the tire tests (blue) are clearly visible along with a third, somewhat weaker mode around 50 nm. The PSD of the tire + disk brake test shows also the small 10 nm mode along with larger particles around 220 nm. This larger mode is known to be assigned to brake wear particles from the literature, where a similar size distribution was found using also elecromobility.^22^ Moreover, two small bumps at 20 nm and 50 nm showing additional tire wear emissions, as they are at the same size range as found for the tire tests.


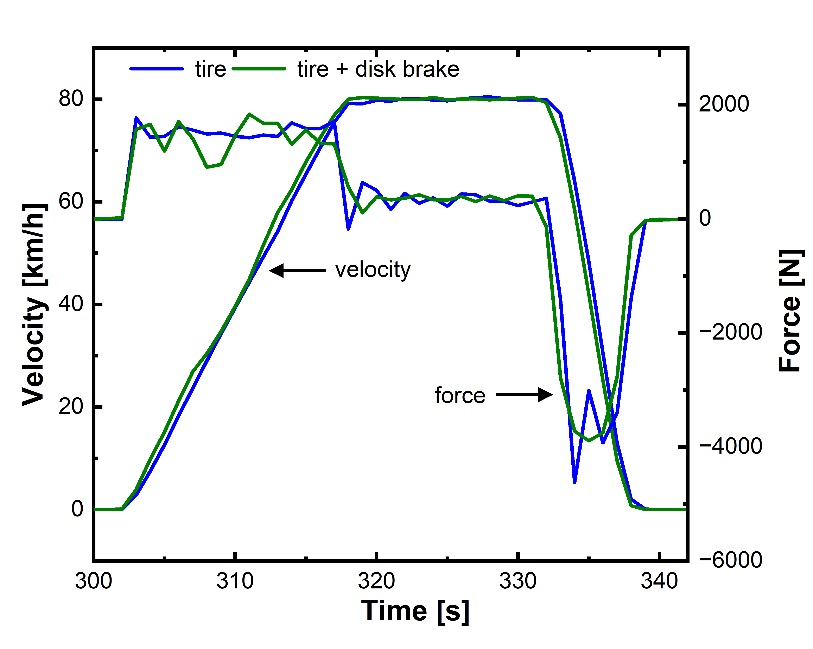


**Fig. S2** Last part of the ZEDU Brake cycle as visualized in Fig. 2


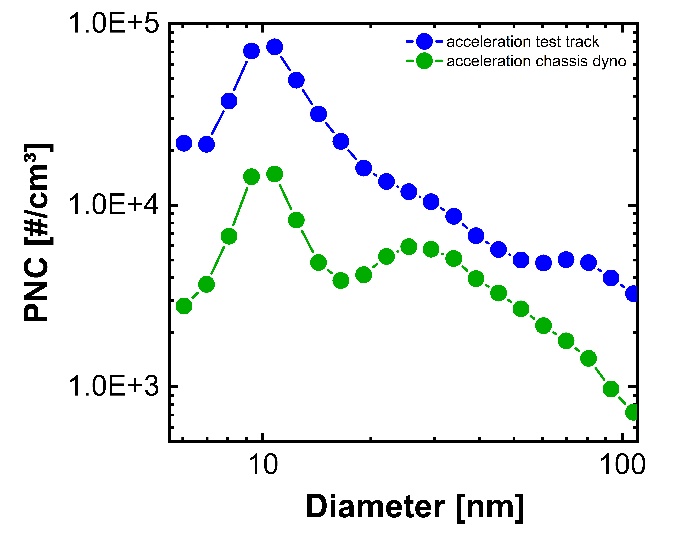


**Fig. S3** PSD derived from EEPS data recorded on the chassis dynamometer (green) and on the test track in Boxberg (blue). The individual spots are averaged values during a period of acceleration (between 1550 s and 1625 s)

The green curve of **Fig. S3** shows the distribution during acceleration and during stop on the chassis dynamometer. As seen from the contour plot a two modal distribution is visible with peaks around 10 nm and 25 nm. The average PSD recorded on the test track is shown in blue. Here the similar small mode with a maximum around 10 nm is visible with more particles in the smallest channel (6 nm) compared to the PSD on the chassis dynamometer. The second mode around 25 nm is only faintly visible and is not separated from the smaller particle mode. Rather there is a single mode between 6 nm and 50 nm. There is also a third, larger distribution with a peak around 70 nm that was not observed on the chassis dynamometer. Our previous experiments showed particles in this size range during cornering and can therefore be assigned to tire emissions from lateral acceleration.


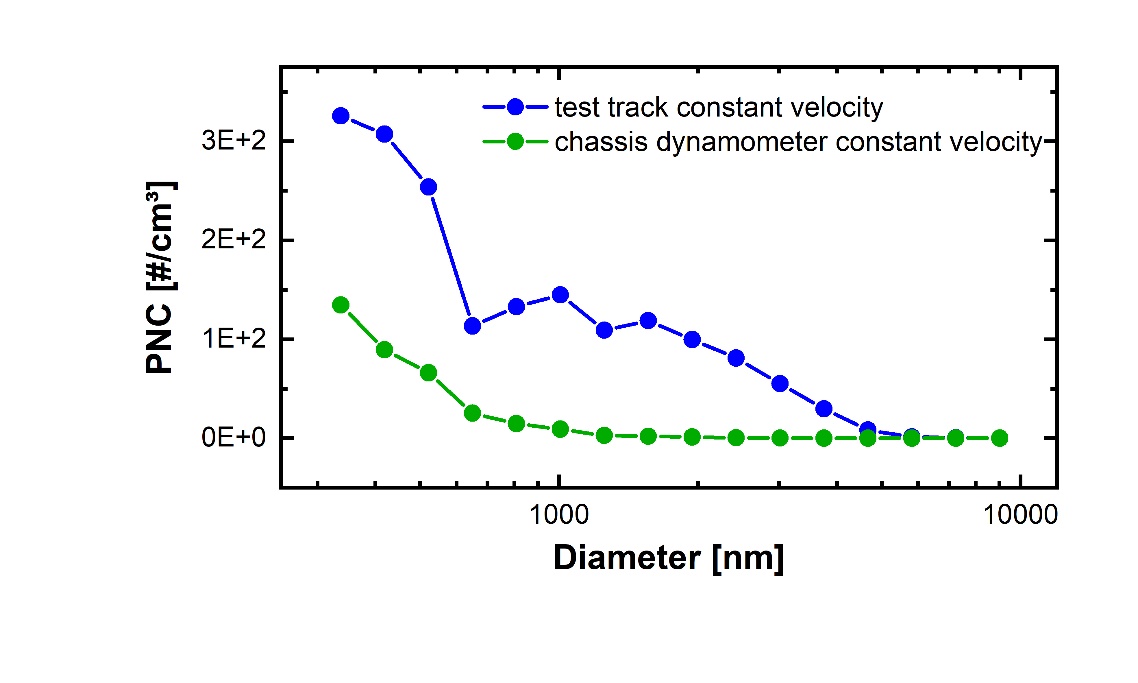


**Fig. S4** PSD derived from OPS data at the sampling position in front of the filter recorded on the chassis dynamometer and on the test track. The values are means recorded between 1664 s and 1730 s at constant velocity

Fig. **S4** shows the two PSDs as average values derived from OPS data at the last constant velocity (between 1664 s and 1730 s). On the chassis dynamometer an almost exponential increase in PNC between 1000 nm and 300 nm is observed. At particle diameters larger than 1000 nm (1 µm) only a flat curve is present. As already seen in the contour plot in Fig. 4 larger particles are appearing on the test track. Here the highest concentration is observed at the smallest 300 nm channel which steeply decreases to 600 nm. However, two additional modes around 1000 nm and 1500 nm are visible. These are either particles that are already present in the environment air and are picked up by the suction, which is also suggested by the higher particle measurements from Fig. 3, or are caused by the changed abrasion mechanism due to the different surface of the road compared to the chassis dynamometer.

**
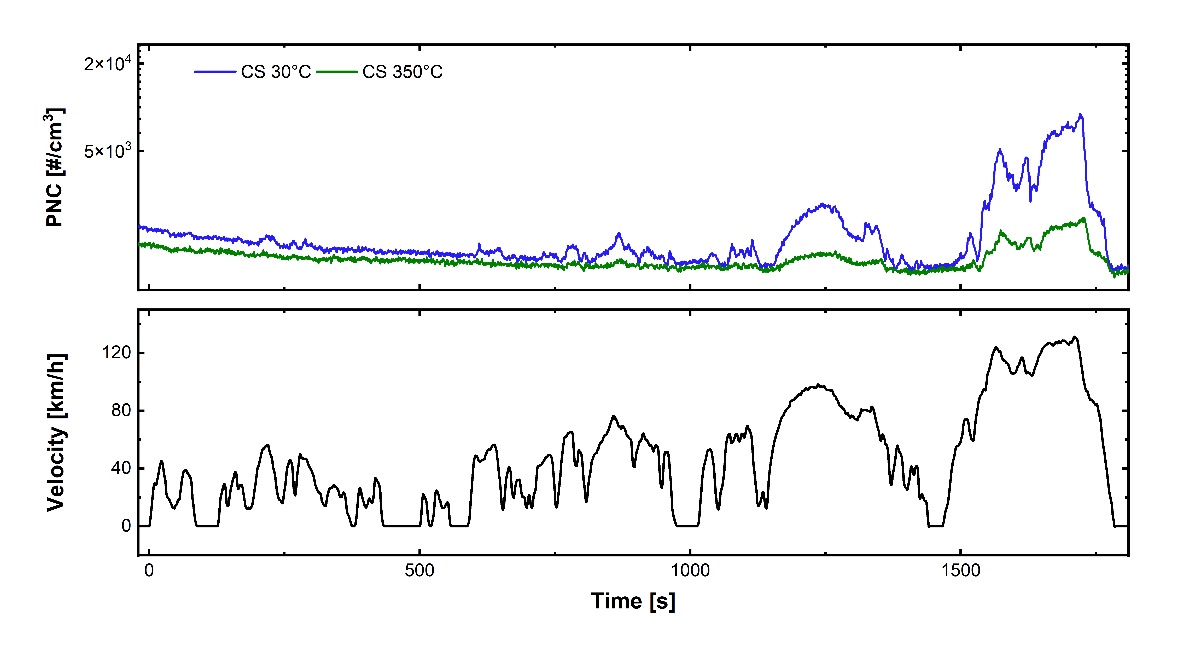
**

**Fig. S5** Comparison of PNC recorded during WLTC with a catalytic stripper at different temperatures.


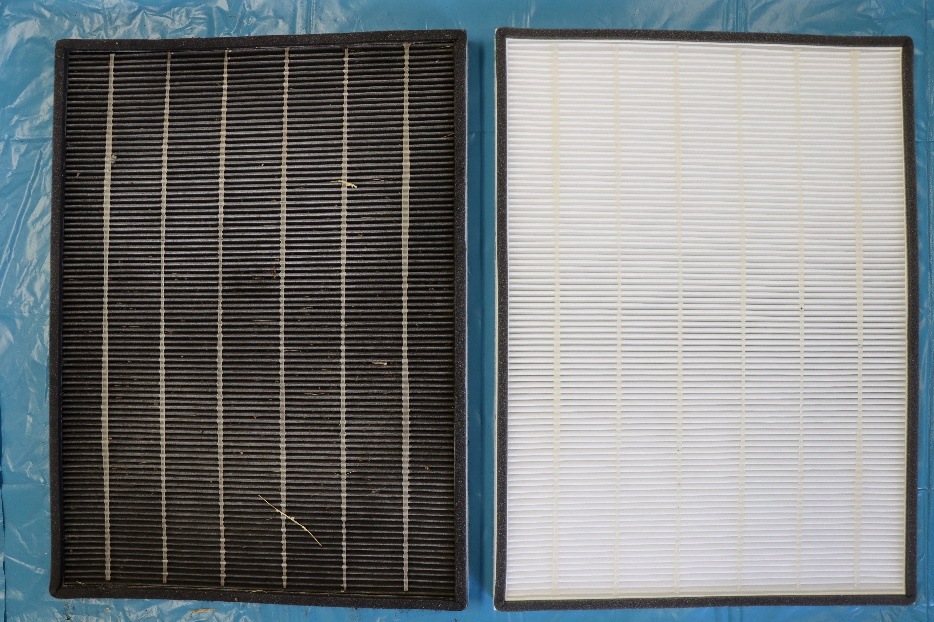


**Fig. S6** Pre-filter of the ZEDU vehicle before (left) and after driving tests on the test track. Size of the filter approx. 57 x 42 x 4 cm

**
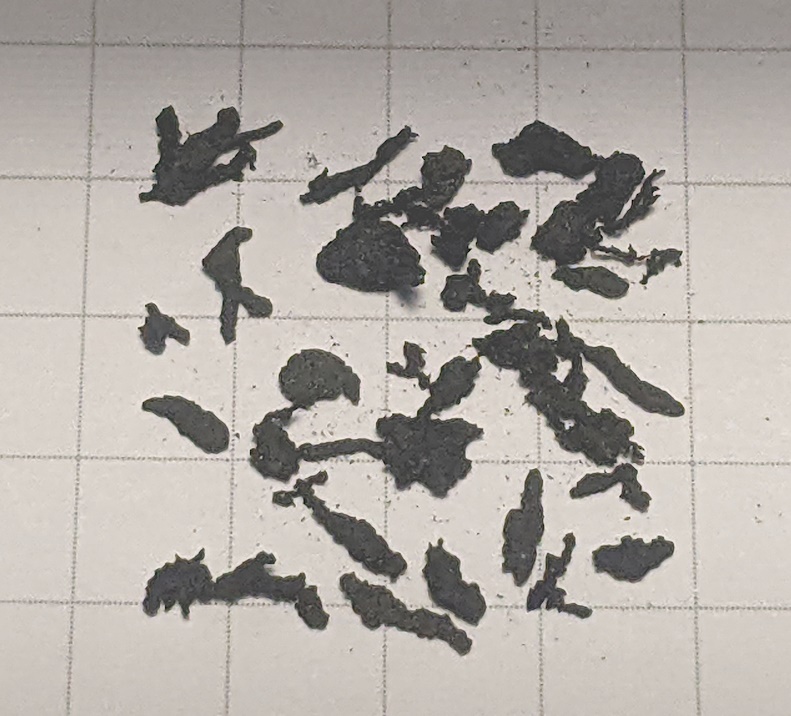
**

**Fig. S7** Larger rubber chunks as collected on test on the test facility in front of the filter. Size of one square is 5mm.
